# The Souvenir Volume of the Wells Celebration

**Published:** 1895-02

**Authors:** 


### THE SOUVENIR VOLUME OF THE WELLS CELEBRATION.

THE chairman of the committee having this report in charge announces on another page that he is ready to receive subscriptions. Those interested in this memorial volume should address Dr. J. D. Thomas, 912 Walnut Street, Philadelphia.

